# Space weather phenomena on heart rate: a study in the Greek region

**DOI:** 10.1007/s00484-022-02382-3

**Published:** 2022-10-13

**Authors:** Maria Papailiou, Sofia Ioannidou, Anastasia Tezari, Dimitra Lingri, Maria Konstantaki, Helen Mavromichalaki, Svetla Dimitrova

**Affiliations:** 1 Athens Cosmic Ray Group, Faculty of Physics, National and Kapodistrian, University of Athens, Athens, Greece; 2 Metaxa Cancer Hospital of Piraeus, Piraeus, Greece; 3 Medical Physics Laboratory, Faculty of Medicine, National and Kapodistrian, University of Athens, Athens, Greece; 4 Space Research and Technologies Institute, Sofia, Bulgaria

**Keywords:** Heart rate, Cosmic ray intensity, Geomagnetic activity, Space weather

## Abstract

Many scientific investigations have focused on how space weather phenomena, taking place in the vicinity of the Earth, may influence different aspects of life on Earth and presumably human health itself. From 2005, the National and Kapodistrian University of Athens has established an important position in the field of these investigations by collaborating with various scientists and Institutes, both international and domestic, in different heliobiological projects. In this work, the Cosmic Ray Group of the National and Kapodistrian University of Athens has co-operated with the medical staff from different hospitals and clinics around the country so as to develop large records of medical data (heart rate) which covers a long time period. These data are analyzed in regard to physical activity, either on a daily basis or on different levels of geomagnetic disturbances and variations of the cosmic ray intensity using the ANalysis Of Variance (ANOVA) and the multiple linear regression analysis. Results suggest that space weather phenomena may be related to heart rate variability, i.e., heart rate is statistically significantly effected either by variations of cosmic rays intensity or geomagnetic activity.

## Introduction

The influence of external factors on the human body is not a matter of the present. Hippocrates already knew that dryness, humidity, and high and low temperatures, in regard to the different seasons of the year, have different effects on human health. The reviews from Persinger (1987), who cites the results of more than 95 authors; Zhadin (2001), who cites the results of research of the last 20–30 years conducted by around 170 Russian researchers; and more recently Zenchenko and Breus (2021), who cite the result of the heliobiological studies of the last 25 years emphasizing on how human physiological parameters may be influenced by geophysical parameters, are undeniable evidence that the influence of space weather phenomena and geophysical variations on human health and well-being is a field of intense, interdisciplinary, international, and ongoing investigation.

A number of more recent studies show that, in addition to animal behavior (Wiltschko and Wiltschko 2005), the activity of the Sun and other related changes in the geophysical environment could affect human health and functions of the human body (Cornelissen et al. 2002; Dimitrova et al. 2019). The normal human condition is affected by changes in environmental factors, which require a series of adaptation reactions from the body and its nervous system. These reactions weaken when various diseases are under discussion (Dimitrova 2006). As it is shown cardiovascular, nervous, and other functional systems respond to changes in geophysical parameters (Zhadin 2001; Cornelissen et al. 2002; Podolská 2021). In most cases, the reactions observed are aimed at adapting the body, to more easily endure these changes and ultimately the biological system to survive in the changed environment. On several occasions, however, such a protective reaction to changes in environmental factors is not observed or is not sufficiently expressed, in which case the living organism is also threatened. The existence of this reaction is especially important in patients or unstable organisms that are organically and emotionally burdened (Dimitrova 2006).

The aforementioned investigations have concluded in the rise of three new scientific fields, biogeomagnetics, clinical cosmobiology, and heliobiology. Biogeomagnetism (Dorman et al. 2001) describes how space weather parameters generally and the geomagnetic field especially may affect the pathological condition of various diseases (Dorman et al. 2001; Stoupel 2002). Results obtained in the laboratory regarding the blood’s sensitivity to solar and GMA strengthen this new field, while the most important results concern diseases of the cardiovascular and nervous systems (Dorman et al. 2001). It is being argued that Forbush decreases (FDs) of CRI are representative of the relationship between geomagnetic disturbances and health parameters, e.g., ischemic attacks, myocardial infarction, and traffic accidents (Villoresi et al. 1994a; b; 1998; Ptitsyna et al. 1996; Dorman et al. 1999). Moreover, during the development of a geomagnetic disturbance, the most important and statistically significant results are observed in the decrease phase of the FDs of CRI.

Moreover, in Breus et al. (2008), the effects of solar and geomagnetic activity on a biological system are being studied. Specifically, the investigation focuses on how biological systems interact with weak (< 1 mT) low-frequency electromagnetic fields and the progress of the possible nonthermal mechanisms of this interaction.

Respectively, clinical cosmobiology is the branch that examines the relationship between the level of physical activity observed in the environment and the frequency of deaths due to heart arrhythmias, myocardial infarction, other cardiovascular diseases, strokes, homicides, suicides, etc. (Stoupel 2006, 2019).

In Podolská (2018), the mortality due to diseases of the circulatory system is being studied in relation to solar activity changes (during solar cycle no 23, and its minima with the unusually low level of solar activity). Solar activity indices, geomagnetic indices, and ionospheric parameters were analyzed in relation to the number of deaths from 1994 to 2011 in the Czech Republic. It was concluded that the number of cardiovascular caused deaths can be best described using the ionospheric parameters rather than the solar indices. Therefore, variations in solar activity and abnormal solar events can cause an indirect response from cardiovascular diseases, through a concentration of electrical charges in the earth’s environment.

Finally, heliobiology or as mentioned in Babayev (2008) cosmobiology or astrobiology examines the effect of solar activity on living organisms and especially on humans (Palmer et al. 2006; Babayev and Allahverdiyeva 2007).

Space weather is determined by physical processes in the Sun and cosmic rays activity. An International project carried out by Russia, Ukraine, and Bulgaria, called “Geliomed” (http://geliomed.immsp.kiev.ua), studies the human health state in relation to geophysical parameters through the cardiovascular system of a group of healthy volunteers. That being so the immediate effect of electromagnetic solar radiation and the influence of solar wind and the interplanetary magnetic field, mediated through geophysical parameters, on the cardiovascular system have been investigated (Samsonov and Manykina 2012).

Ozheredov et al. (2017) have investigated the earth’s weather conditions such as pressure, temperature, and humidity which correspond to days when the human body is the most sensitive to changes in the geomagnetic field variations and when it reacts by statistically significant increase (or decrease) of a particular physiological parameter. Results show magneto-sensitivity of systolic and diastolic blood pressure and heart rate of healthy young subjects for three weather areas (combinations of atmospheric temperature, pressure, and humidity).

Sasonko et al. (2019) have presented results concerning the effect of local atmosphere status and space weather on 12 healthy volunteers and 15 patients suffering from arterial hypertension using a 24-h electrocardiogram monitoring for the time period from November 23, 2016, until March 29, 2017, when 4 moderate and 11 minor magnetic storms occurred. In the periods of weak frost and intense precipitations (snow or rain with snow), the combination of the rather high horizontal component of the magnetic field with elevated atmospheric pressure and humidity resulted in abnormal RR, PR, and QT intervals of the electrocardiogram.

On the other hand, Mattoni et al. (2020) have studied the correlation between heart rate variability and geomagnetic/solar activity for 20 healthy participants by measuring their heart rate variability for a 30-day period and solar activity during this period. Results showed that the effects of geomagnetic and solar activity are (if present) most likely of very small effect.

Over the last years, the Cosmic Ray Group of the National and Kapodistrian University of Athens (NKUA) has intensively worked on how the human physiological state may possibly be effected by space weather phenomena and has published a significant number of scientific papers presenting the results of these investigations (Papailiou et al. 2009, 2011, 2012; Mavromichalaki et al. 2012, 2017, 2021; Giannaropoulou et al. 2014; Galata et al. 2017). In this work, an attempt to present a synopsis of the studies on heart rate (HR) variations that have been conducted by the NKUA in Greece so far is being made. It should be mentioned that essential for these studies have been the collaborations with many scientific groups and institutes, both science and medical, which resulted in the development of a large database of physical parameters (CRI, geomagnetic indices, etc.) and medical data (mean HR, pulse, etc.). Moreover, the medical data refer to the time period from 2011 to 2018 (from the Hippocratio General Hospital, Athens; the Cardiology clinics of Nikaia General Hospital, Piraeus and the Heraklion University Hospital, Crete). Furthermore, the medical data in the aforementioned database were organized according to age, health state/health conditions, gender, etc. In this way, there is an opportunity of examining how volunteers of different ages, with or without heart conditions, on medication, etc., respond to different levels of geomagnetic and cosmic ray activity.

## Data and method

### Holter electrocardiogram method

This method consists of the continuous 24-h electrocardiogram (ECG) recording on tape or even electronic memory, as well as the subsequent analysis of this data by a computer. With the Holter method and while the examinee continues to perform his daily activities, various cardiac events can be assessed, which often occur sporadically so that it is unlikely to be recorded on a simple ECG. Such cases are mainly arrhythmias, but also myocardial ischemia to a lesser extent.

The continuous ECG recorder is a small portable device the size of a small tape recorder with 5–7 wires, which are connected to the chest of the examinee with self-adhesive tapes. The device records 24 h of two or three abductions on the ECG and operates on batteries. The examinee constantly carries it with him, continuing his daily activity. Then, after 24 h, the tape is placed on a special computer, which analyzes its data and takes the result in about 20 min.

The analyzer is a device with which the ECG recorded on the magnetic tape is converted into a real ECG, which is projected on the screen at a speed of 30, 60, or 120 times faster than the real one. The examiner monitors on the screen every variation in the rhythm and morphology of the various changes of the ECG and has the ability to record the points of interest with the speed of the usual ECG. The resulting report lists the patient’s arrhythmias, what the patient’s rhythm was when he pressed the onset button, and any drop in the ST interval. The latter is useful for the diagnosis of silent ischemia (myocardial ischemia without symptoms).

Continuous recording of the ECG (Holter) is useful mainly in two situations: (a) detection of arrhythmias and (b) myocardial ischemia. The method is more reliable in the study of arrhythmias. It is possible to determine the type of arrhythmia or arrhythmias as well as their severity. Also, in patients with a permanent pacemaker, any pacemaker malfunction can be assessed, so that the necessary actions can be taken. Antiarrhythmic therapy can also be evaluated by comparing Holter tests before and after medication. In the diagnosis of myocardial ischemia, there is less reliability compared to the plain ECG. In regard to the Holter recording, many times, factors such as posture, climatic conditions, and possibly geomagnetic storms can affect the state of the human cardiovascular state, causing ischemia or other cardio symptoms. However, there are some characteristics that can help an experienced doctor make a proper assessment. In general, however, as with other tests, in order to make a diagnosis, in addition to the Holter test, it is usually necessary to make a comprehensive assessment of all the data (clinical and laboratory) that exist for the patient.

### Geomagnetic and cosmic ray activity data

Geomagnetic index Dst data were used in order to analyze the GMA during the time period under examination. The World Data Centre for Geomagnetism, Kyoto, supplies online real-time data through via website http://wdc.kugi.kyotou.ac.jp/dst_realtime/index.html.

Furthermore, the hourly, pressure-corrected data of the hadronic component of the CRI used in this study were available through the Athens Neutron Monitor Station (A.Ne.Mo.S.) of the Faculty of Physics of the National and Kapodistrian University of Athens. This neutron monitor station (Super 6NM-64, cut-off rigidity of 8.53GV), which operates since November 2000, provides real-time data with time resolution of 1 h, 1 min, and 1 s through the Internet (http://cosray.phys.uoa.gr) (Mavromichalaki et al. 2001, 2005) and transfers these data on a 1-min basis to the European High-resolution Neutron Monitor Database (NMDB) (http://www.nmdb.eu).

The relation $\mathrm{CRI}=\frac{{\mathrm{CRI}}_{\mathrm{obs}}-{\mathrm{CRI}}_{\mathrm{aver}}}{{\mathrm{CRI}}_{\mathrm{aver}}}$, was used in order to normalize the CRI data, where ${\mathrm{CRI}}_{\mathrm{obs}}$ is the observed CRI value and ${\mathrm{CRI}}_{\mathrm{aver}}$ is the average value relating to the examined period.

The analysis of HR variations in relation to CRI variations consists of three different steps: (a) during cosmic ray activity, i.e., mainly during Forbush decreases; (b) during quiet periods of CRI, i.e., no registered events, even though no events are registered CRI has also a diurnal variation, e.g., a 24-h variation with a minimum value around 6a.m. and a maximum value on 6p.m. By plotting the time profiles for HR/CRI for each patient, we examine this diurnal variation; and (c) during different levels of cosmic rays activity.

### Statistical method

The statistical methods of analysis of variance (ANOVA, http://www.statisticssolutions.com/manovaanalysis-anova/) (Papailiou et al. 2012; Dimitrova et al. 2013) and of multiple linear regression analysis were applied, so as to determine the effect of cosmic rays and geomagnetic disturbances on the human physiological parameters (i.e., HR). A univariate analysis was conducted alongside, in order to evaluate the importance of the negative confounding factors. Moreover, calculated and discussed were the respective levels of significance (*p*-values).

While applying the regression models, HR is considered as the dependent variable, while CRI, Dst index, age, gender, and a unique patient identifier (to control for inter-patient variability) are regarded as the independent variables (Galata et al. 2017). Furthermore, for the statistical analysis, the statistical packages SPSS (IBM SPSS Statistics 20) and STATISTICA (ver. 10, Stat-Soft INC., 2011) were also used.

## Data for the time period 2011–2018

In this study, patients of the Hippocratio General Hospital in Athens, the Cardiology clinics of Nikaia General Hospital in Piraeus, and the Heraklion University Hospital in Crete, Greece, were evaluated from 2011 to 2018. A total of 1318 individuals were examined. A Holter ECG was used in order to record the hourly HR of the patients.

The time period under investigation includes both the ascending and the descending phase of the solar cycle 24 (maximum in the year 2014), which lasted until 2019. Therefore, it was decided that the sample was imperative to be analyzed not only as a whole for the entire time period under examination, but also in parts throughout the two phases of the solar cycle and on specific events and FDs. Herein, the most interesting results concerning the ascending phase of the solar cycle 24 and the related FDs are being presented. Moreover, some preliminary results for the descending phase of the solar cycle are also included.

### Data analysis for the time period 2011–2018

#### Ascending phase of solar cycle 24 (2011–2014)

The group under examination included 482 individuals. The 57% (275) were men, the 41% (198) women, and the 2% (8) individuals with no further known details. There is not a noteworthy variance concerning the HR mean values for the men and women under investigation (mean HR_men_: 69 ± 12 bpm, mean HR_women_: 71 ± 12 bpm). Nevertheless, it is important to report that the response of the HR of men and women during GMA and CRI variations varies, as described in other investigations (Papailiou et al. 2009).

A detailed and descriptive analysis of the data for the time period 2011–2014 (covering the ascending phase of solar cycle 24) has been introduced and published in Galata et al. (2017). Therefore, herein, the most interesting results of this investigation are being summarized and briefly presented.

In total, for the abovementioned time period, 10,720 measurements of CRI, Dst index, and HR were recorded and analyzed. The total CRI values and the GMA data were divided into levels 0, 1, 2, 3, and 4 in accordance with the observed CRI variations and geomagnetic disturbances (Galata et al. 2017). For this study, a geomagnetic storm corresponds to Dst index less than − 100nT (Papailiou et al. 2011). Finally, for every level of activity, the relative mean HR values were estimated as well. A suchlike classification technique has been introduced in earlier works, e.g., Papailiou et al. (2012) and Mavromichalaki et al. (2012).

In a bivariate analysis approach, CRI was correlated to HR (− 0.024 and *p*-value 0.011) as well as Dst index (− 0.024 and *p*-value 0.012). After applying the multiple linear regression model, adjusted for daytime and individual patient, an independent association between CRI and Dst index and HR was established. A negative correlation was revealed for both CRI and Dst index to the HR with a regression coefficient − 0.025 (95%CI: − 0.179, − 0.025) and *p*-value 0.009 and with a regression coefficient − 0.022 (95% CI: − 0.035, − 0.003) and *p*-value 0.023, respectively. Moreover, for women, in comparison to men, the correlation of HR with GMA and CRI variations appears to be much greater. Specifically, results revealed a double percentage for women (40%) in relation to men (20%) with a correlation coefficient over 0.25 (Galata et al. 2017).

The ANOVA analysis approach revealed that HR and CRI were statistically significantly negatively correlated (*p* < 0.01), whereas HR and Dst index variations were significantly associated (*p* = 0.05). The mean HR values for the five separate CRI and Dst index levels, respectively, were studied. As it is shown, when the mean CRI is increased (from level 0 to level 5), the HR decreased. Furthermore, for geomagnetic disturbances of low intensity, e.g., low levels of Dst index, the HR variations were minor.

A number of FDs (but not strong events) of CRI were registered, during the time period under examination. The FDs amplitude ranged from 2 to 12%. The largest event was registered during March 2012. This FD is related to an X-ray flare (X5.4) registered on 07/03/2012 at 00:02UT in AR 1429. It was followed by a halo CME recorded on 07/03/2012 at 00:24UT by SOHO/LASCO (cdaw.gsfc.nasa.gov) with a linear speed of 2684 km/s. The shock arrived at the Earth on 08/03/2012 at 11:05UT, when a sudden storm commencement (SSC) was recorded. The time interval from March 5 to March 21, 2012, was isolated and analyzed separately, because a FD with amplitude of 11.7% was registered by the neutron monitors (Galata et al. 2017). A geomagnetic storm occurred on 09/03/2012 (Dst index =  − 131nT). For the abovementioned period of time, 912 HR observations obtained by Holter were studied by applying a multiple linear regression analysis. HR and the respective CRI values were statistically significant negatively correlated (regression coefficient: − 0.138, *p* < 0.01) for the 38 patients under investigation.

**Fig. 1:**
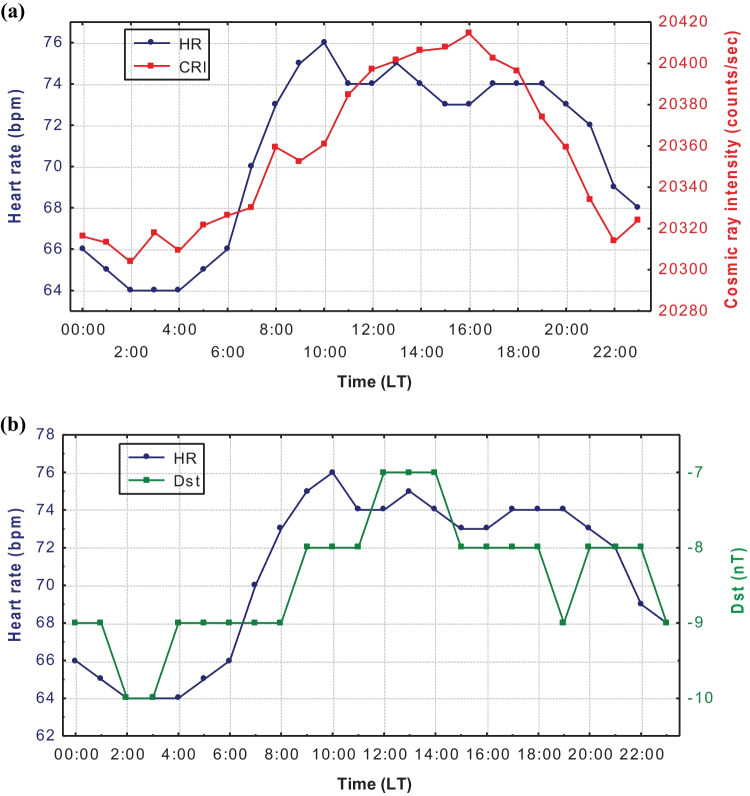
The daily distribution of the mean hourly values of HR and CRI (**a**) and Dst (**b**) from July 2011 to April 2013

The whole sample of the medical data was analyzed diurnally as well, for each individual separately. In this analysis, it is concluded that, while in some occasions the hourly values of HR display a daily variation similar to CRI, in others it seems to be the exact opposite. Furthermore, the distribution of the CRI and Dst variations and the mean HR (24-h Holter measurements) for all cases was plotted for the time period under examination (Fig. 1a,b respectively). In addition to the expected CRI diurnal variation, a similar behavior in HR values is also indicated (Galata et al. 2017).

#### Descending phase of solar cycle 24 (2014–2018)

**Fig. 2:**
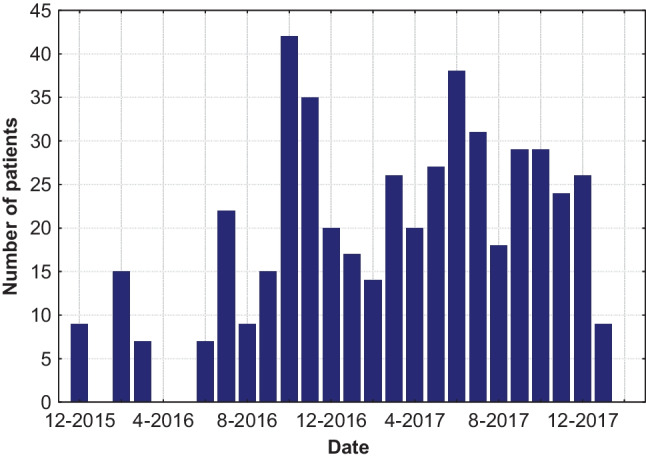
Distribution of patients for the descending phase of solar cycle 24 (2015 to 2018)

For the time period 2015–2018, 9429 measurements were registered and processed. These measurements refer to CRI, Dst index, and HR data. Figure 2 displays the number of patients’ distribution for the time period 2014–2018.

Cosmic ray data were divided into five levels (0, 1, 2, 3, 4) representing events of different intensities as shown in Table 1. Similarly, the Dst index values were, also, separated into levels (0, 1, 2, 3, 4) introducing GMA of various intensities. Finally, Table 1 is completed by the mean HR values for every level, corresponding to the time period under investigation.

**Table 1:** The levels of the CRI and the GMA (Dst index) as organized for the ANOVA analysis. The corresponding number of measurements and the mean HR values are provided

Levels	Intervals	Number of measurements	Mean HR (bpm)	CI (95%)
CRI				
0	CRI ≤ − 3%	39	79.85	74.87–84.82
1	− 3% < CRI ≤ − 1%	1103	74.40	73.15–75.64
2	− 1% < CRI ≤ 0%	3410	73.32	72.60–74.04
3	0% < CRI ≤ 1%	3957	74.15	73.45–74.85
4	CRI > 1%	920	73.50	72.04–74.96
Dst index				
0	Dst ≥ 0nT	2459	72.19	71.38–73.01
1	− 20nT < Dst < 0nT	5005	74.82	74.18–75.46
2	− 50nT < Dst ≤ − 20nT	1764	73.48	72.51–74.45
3	− 100nT < Dst ≤ − 50nT	187	72.26	69.60–74.91
4	Dst ≤ − 100nT	14	79.50	70.55–88.45

In a bivariate analysis approach, CRI was statistically significantly correlated with HR (0.039 and *p*-value 0.003), while Dst index was not statistically significantly correlated with HR (0.019 and *p*-value 0.092). The ANOVA analysis indicated that on average HR is different between the Dst index groups (*p* = 0.002); no statistically significant results for the respective CRI level analysis (*p* = 0.274) were observed.

**Fig. 3:**
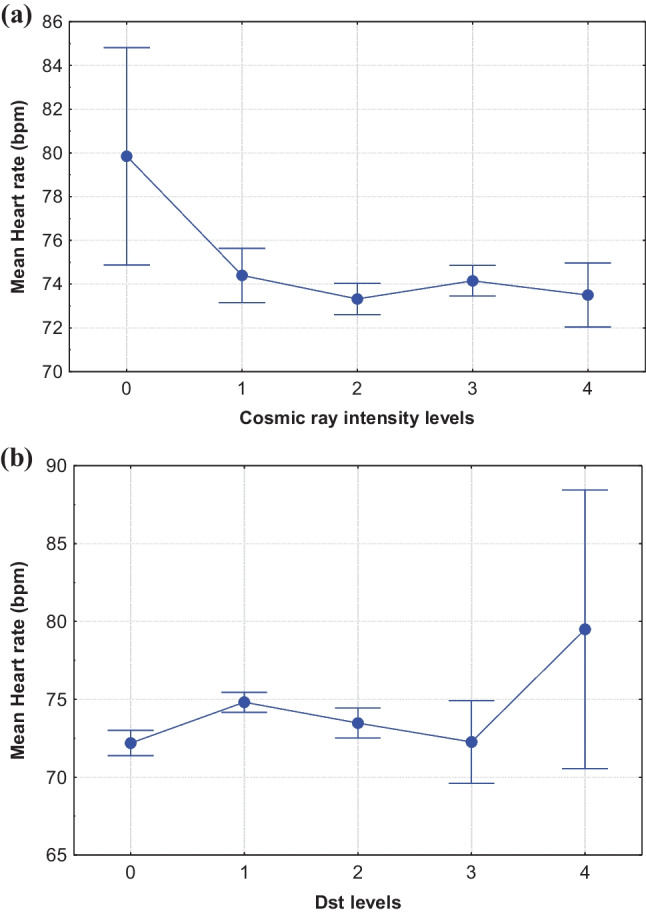
Mean HR values distribution for the five CRI levels (**a**) and Dst index levels (**b**)

Figure 3 shows the variation of the mean HR for the five levels of the CRI and GMA (Dst index), respectively. As it is presented, when the mean CRI is increased (from level 0 to level 4), the mean HR decreased (Fig. 3a). Furthermore, high GMA (i.e., high levels/low values of Dst index) corresponds to the maximum value of mean HR (Fig. 3b).

Table 2 shows a summary of the statistical results of this study for both time periods, i.e., the ascending and the descending phase of solar cycle 24.

**Table 2:** Statistical results for the time periods 2011–2014 and 2014–2018

1. Bivariate analysis		
	Correlation coefficient r	*p*-value
2011–2014		
CRI-HR	− 0.024	0.011
Dst-HR	− 0.024	0.012
2014–2018		
CRI-HR	0.039	0.003
Dst-HR	0.019	0.092
2. Multiple linear regression		
	Regression coefficient	*p*-value
(a) Quiet period		
CRI-HR	− 0.025	0.009
Dst-HR	− 0.022	0.023
(b) Disturbed period/FD during March 2012		
CRI-HR	− 0.138	< 0.01

**Fig. 4:**
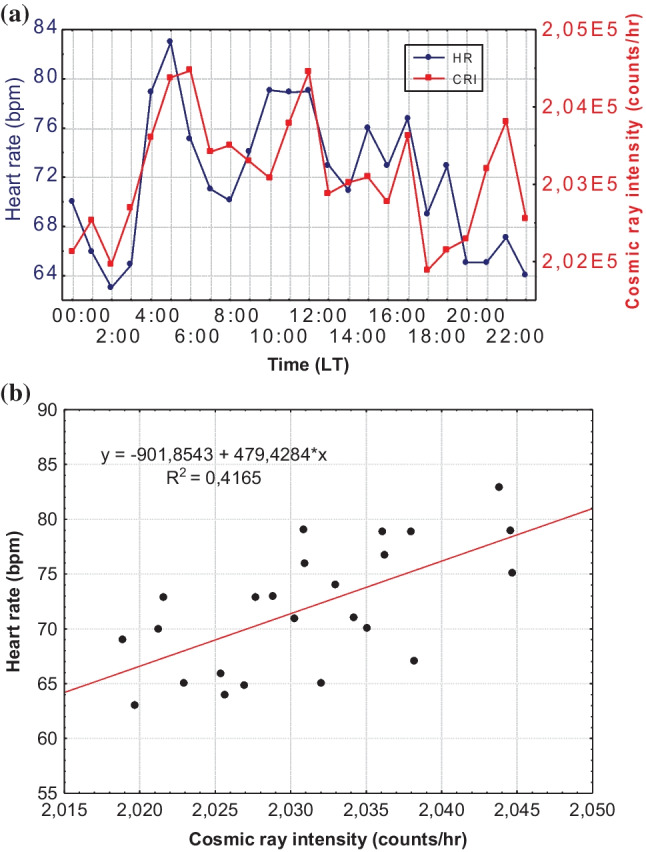
Hourly values of CRI and HR (**a**) and bivariate correlation diagram of CRI and HR (**b**) for a male patient on April 28, 2017

**Fig. 5:**
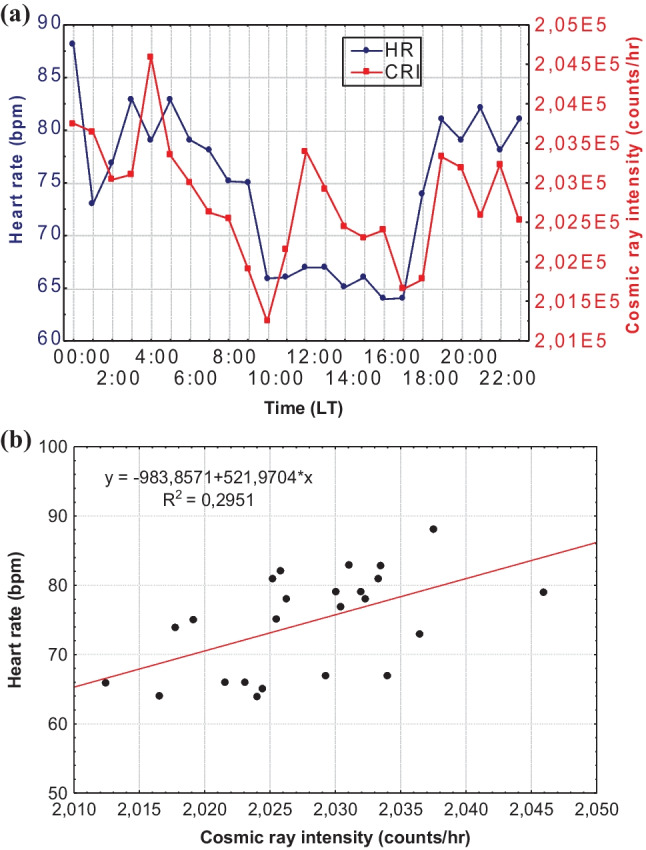
Hourly values of CRI and HR (**a**) and bivariate correlation diagram of CRI and HR (**b**) for a female patient on September 15, 2016

Moreover, the medical data were also analyzed diurnally, separately for every patient/volunteer. Two examples of the daily distribution of HR and CRI and the corresponding bivariate correlation diagram for two patients of the sample are presented below. Figure 4 refers to a male patient on April 28, 2017 (*R*^2^ = 0.4165), and Fig. 5 refers to a female patient on September 15, 2016 (*R*^2^ = 0.2951).

## Discussion and conclusions

Human health can be affected by endogenous (nutrition, stress, quality of life, etc.) as well as exogenous factors (Unger 2019). For example, human health depends on meteorological weather (Khabarova and Dimitrova 2009), radiation exposure, exposure to chemical substances, etc. Even though separating the effect of each of these is a difficult problem to solve, various data analysis approaches and methodologies can help differentiate the effect of each factor. However, during the last decades, the emergence and development of a new scientific field has also played an important role in determining the human health conditions, and that is Space weather and its phenomena.

The Athens Cosmic Ray Group of the NKUA has investigated the effect of space weather and more precisely of solar phenomena, geomagnetic disturbances, and CRI variations on the human state (both physiological and psychological). Therefore, over the last few years, it has earned a leading role in this kind of studies. In general, four projects, concerning data from different geographical regions, were carried out by cooperating with scientific teams from various countries (Baku, Azerbaijan; Kosice, Slovakia; Tbilisi, Georgia; Piraeus, Greece).

Data on mean heart rate, RR intervals, arterial systolic and diastolic pressure, or the number of occurrence of different types of cardiac arrhythmias, etc., were analyzed in regard to GMA, manifested by the geomagnetic indices Ap and Dst and CRI variations (FDs and CRI enhancements). Based on the time resolution of the medical data (diurnal or annual data), the aforementioned investigations cover various periods of time and refer to either chosen healthy persons or random individuals. It should be mentioned at this point that neither neutron monitors nor the Holter electrocardiogram device is influenced by changes in physical conditions during solar storms.

As a result of these investigations, geomagnetic disturbances and CRI variations seem to have an effect on the physiological state of the human organism. Furthermore, geomagnetic storms and FDs of the CRI result in variations of the human physiological parameters. More precisely, high GMA and strong CRI decreases were related to HR, RR interval, SP, and DP variations (Papailiou et al. 2011, 2012; Mavromichalaki et al. 2012, 2021; Galata et al. 2017), whereas the connection (correlation or anti-correlation) of solar, geomagnetic, and CRI variations to the number of occurrence of different types of cardiac arrhythmias was established in a number of studies (Giannaropoulou et al. 2014; Mavromichalaki et al. 2017, 2021).

With this work, the NKUA team attempted an analysis on a nationwide scale by using a large volume of measurements. As a result and as is presented herein, an extensive database of medical information is assembled with the co-operation of medical staff from different hospitals and clinics around the country. The aforementioned database covers a wide time period making it possible to examine the behavior of human physiological parameters through geomagnetic and solar activity of varied intensity (from quiet conditions to extremely high activity conditions) and moreover during geomagnetic storms and FDs. Moreover, these data have been also analyzed on a daily basis with interesting results concerning mean HR diurnal variations in relation to diurnal CRI variations. HR, as many other cardiovascular variables, including blood pressure, etc., demonstrate a circadian rhythm, i.e., an oscillation of a physiological process over a 24-h period. In the present study, each case independently has been analyzed on a daily basis, in order to examine whether HR follows the CRI diurnal variation (24-h variation with a minimum value around 6a.m. and a maximum value on 6p.m). It is noticed that even though in some cases, HR and CRI are positively correlated in other cases the parameters seem to have an opposite behavior. This is a finding that is worth mentioning in the results, but requires further investigation.

Generally, it can be argued that environmental physical activity may be associated to HR variations, since both CRI decreases and geomagnetic storms have a statistically significant effect on HR. The ultimate goal is to have solid and irrefutable results on the effect of the phenomena of space weather on human health.

## Data Availability

The datasets generated during and/or analyzed during the current study are not available to the public.
